# Site Selection Criteria for Sheltering after Earthquakes: A Systematic Review

**DOI:** 10.1371/currents.dis.17ad1f98fb85be80785d0a81ced6a7a6

**Published:** 2014-08-29

**Authors:** Ahmad Soltani, Ali Ardalan, Ali Darvishi Boloorani, AliAkbar Haghdoost, Mohammad Javad Hosseinzadeh-Attar

**Affiliations:** Department of Disaster Public Health, School of Public Health, Tehran University of Medical Sciences, Tehran, Iran; Department of Disaster and Emergency Health, National Institute of Health Research, Tehran University of Medical Sciences, Tehran, Iran; Department of Disaster & Emergency Health, Iran's National Institute of Health Research; Department of Disaster Public Health, School of Public Health, Tehran University of Medical Sciences, Tehran, Iran; Harvard Humanitarian Initiative, Harvard University, Cambridge, Massachusetts, USA; Department of Remote Sensing & GIS, University of Tehran, Tehran, Iran; Research Center for Modeling in Health, Institute for Future Studies in Health, Kerman University of Medical Sciences, Kerman, Iran; Department of Disaster Public Health, School of Public Health, Tehran University of Medical Sciences, Tehran, Iran

## Abstract

Objective: Proper shelter site selection is necessary for long-term welfare of earthquake affected people. This study aims to explore the criteria that need to be considered after earthquakes. Methods: Through a systematic review, 273 articles found that were published till April 2014. Among these, seven articles have been selected and analyzed for the criteria that they introduced for sheltering site selection after earthquakes. Results: Out of 27 proposed criteria, accessibility and proximity to homes of affected people were stressed in all the papers. Moreover, seven other criteria were the same in most of the papers including suitable size, suitable distance from hazardous areas, geological hazards and land slope, suitable distance from medical centers, water supply and Security. We categorized all the mentioned criteria in six main categories. Size and location, disaster risk reduction, relief and rescue facilities, feasibility of the site, environmental and social aspects are the main categories. Conclusion: Selection and applying proper criteria for shelter site selection after earthquakes is a multi-disciplinary task. The decision needs relevant models and/or tools. Geographic Information System (GIS) is a useful tool for this purpose. Key words: Disaster, earthquake, shelter, site selection, systematic review

## Introduction

Earthquake, as an unavoidable hazard, results in death, injury, disruption of social and livelihood 1. Moreover, earthquakes cause people homeless because of destruction of houses, whether completely or partially that leaves houses unsafe and non-usable. From 2000 to 2013, over 12 million people became homeless worldwide because of earthquakes^2^.

Sheltering which is a priority for relief organizations, denotes the activity of staying in a place aftermath a disaster where daily routines are suspended. Shelter is one of the primary needs of the affected people in the post-earthquake phase^3^^,^ 4. Similar to the other reconstruction and rehabilitation processes, settlement of the affected people should be undertaken within a long-term strategy^3^^,^ 5. This strategy could be different in various disasters and communities. In an ideal type which is applied in the United States, it includes four phases. These are emergency sheltering, temporary sheltering, temporary housing and permanent housing^4^^,^ 6.

Emergency shelters are generally for one or a couple of days after the event. This situation usually does not need extensive preparation of food or other services and may be a public shelter, motel, hotel room, friend’s house, tent, trailer, camper, or a plastic sheet. Temporary shelters are for longer periods after a disaster that is few weeks, and can be tent, camp or a public mass shelter. Finally, housing which could be divided into temporary or permanent phases; denotes the return to normal daily activities^4^.

Shelter strategy in some communities differs from this and comprises three phases. Here emergency sheltering is not limited to one or a couple of days and may take several weeks^7^^-^ 9.

It is needed to have various sheltering programs in place. They could be activated in different situations depending on the types of damages and available resources^10^. If not planned, choosing proper site for emergency or temporary shelters will be arranged within a limited time after a disaster.

Moreover, lack of proper criteria for site selection can be led to undesirable consequences or people deny accepting the site^1^. Some consequences include subsequent secondary disasters, lack of safety, cultural or climatic inappropriateness, social problems, delays linked to the procurement of shelters, finding sites, and lack of organizational services^1^^,^ 11.

Some available texts such as the Sphere project present the minimum standards of sheltering in disasters^12^. But the question is “which criteria can be applied for site selection of emergency or temporary shelters in an earthquake affected area?”

With emphasize on the earthquake, this paper aims to show which criteria should be considered for selecting an emergency or temporary shelter site. The findings of this research would help the emergency planners to settle the homeless people in safe and accepted shelters following earthquakes.

## Method

**Databases and Search Strategy**

This study is a systematic review and reports according to Preferred Reporting Items for Systematic Reviews and Meta-Analyses (PRISMA) guideline. The search for scholarly articles was conducted in April 2014 and covered the articles that were published by the end of April 2014 without any limitation on publication year.

We searched PubMed, Scopus, ProQuest Research Library, Ovid,Science Direct, and Google Scholar as our databases without any limitation.

The search terms and keywords were selected after consulting with the relief experts and disaster management researchers. The main search terms applied in three parts: 1) keywords about sheltering and housing such as shelter, sheltering, settlement, resettlement and housing, 2) "Earthquake" as our main subject, 3) keywords about site selection which were site, site selection, site selecting, locate, location, locating and site mapping.

Medical Subject Headings (MeSH) from PubMed used to adjust and control the terms and search the databases even in databases that do not use MeSH to index articles.

In addition, we used the search strategy of the PubMed as a model for search all databases. So our search strategy was as follows: Earthquake* AND (shelter* OR settlement OR resettlement OR hous*) AND (site OR site select* OR locat* OR site map*).

**Inclusion and exclusion criteria**

The research team applied the following criteria for inclusion and exclusion of papers:

Inclusion criteria:

- Articles in the field of earthquake

- Articles about sheltering of affected people

Exclusion criteria:

- Non-English articles

- Articles about temporary or permanent housing

- Articles irrelevant to the subjects of site selection

***Studies selection***

The first author scanned titles of all identified papers for inclusion criteria. The studies that met both of the inclusion criteria selected for the next stage. Included papers selected to review the abstracts. In this stage, the studies that met one of the exclusion criteria excluded. He also excluded duplicated papers.

Full texts of all selected articles scrutinized for any model or decision making criteria to locate shelters, or addressing site selection criteria by first and second authors. They reviewed selected articles to define which one had answered to study question and presented some criteria for sheltering site selection after earthquakes? This stage led to final included articles.

For disagreements in this stage, they had consultation with third and fourth members of the review team. Quality assessment of included studies to evaluate method and data gathering process was also done by reviewers. They also were asked to consider any risk of bias.

**Data analysis**

Data extraction of selected articles performed using a checklist created by the authors. This list included the first author, country of the first author, year of publication, shelter type, selected criteria, definition and source of selected criteria, study aim and method.

Extracted data presented as descriptive and thematic analyses. Descriptive analysis used to show the characteristics of selected studies and presented criteria. The thematic analysis was done to define importance of relevant criteria and categorize them. These categories could be used in developing needed models.

## Results

Our search resulted in 273 potentially relevant articles. After reviewing abstracts and removing the duplicates, 26 articles met the inclusion and exclusion criteria and found potentially relevant to the aim of study. Full text review of these articles led to seven articles (Figure1).

**Figure:**
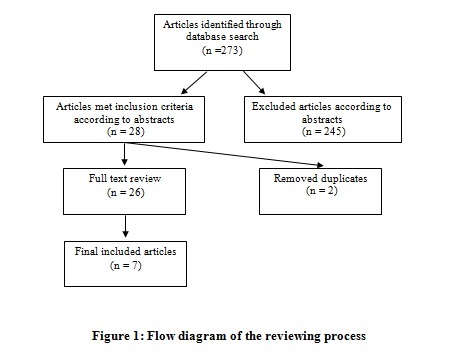

Table 1 summarizes the selected articles for the study variables. The selected studies were conducted from 2011 to 2014 on areas with historical and present earthquake threats.

Most studies aimed to propose proper and systematic models for sheltering site selection after earthquakes. We had little risk of bias for selected studies because of the subject essence.

**Table 1 - Summary of the selected articles for the study variables:** *Multi attribute decision making **Analytic Hierarchy Process *** Technique for order preference by similarity to ideal solution

1th Author	Country	Year	Study Objective	Method	Source of selected criteria
Kilci^13^	Turkey	2014	Developing a methodology for selecting shelter’s site location	Weighting criteria and developing mathematical model	Literature review and Turkish Red Crescent Function
Omidvar^1^	Iran	2013	Proposing a model for appropriate and systematic site selection	Using a geographical information system and MADM^*^	Litrerature review and the options of experts
Jianyu^14^	China	2012	Proposing a model for appropriate and systematic site selelction	Using AHP^##^, entropy methods and TTOPSIS^###^ method	Literature review
Liang^15^	China	2012	Help to decide on shelter locations in small and medium-sized cities	Building covering model and case study	Literature review
Cheng^16^	China	2012	Selecting appropriate and reasonable indicators for emergency shelter	Using AHP	Literature review and the options of experts
Jianyu^17^	China	2011	Establishing an index system for evaluation of emergency shelter site	Using AHP, comprehensive fuzzy evaluation method	Literature review
Qiang^18^	China	2011	Proposing a decision-making tool to support in recovery and reconstruction programs	Lesson learned presentation	Field investigations and observations, as well as experts structured interview

The terms of emergency and temporary sheltering were used in four and three studies respectively. Evaluation of the processes showed that both of them note temporary phase of the United State strategy.

The selected articles addressed 27 criteria in total, for site selection of emergency and temporary shelters following earthquakes (Table 2). Accessibility to the site and proximity to the homes of affected people were proposed in all the papers. Suitable size, suitable distance from hazardous areas and geological hazards, low land slope, proximity to medical centers, water supply and security were the other most common criteria.

**Table 2 - Criteria of shelter site selection following an earthquake:** 

Main Category	Criteria	Definition	Number of artticles	References
Size and Location	Suitable size (population density)	Refers to both the general area and effective refuge area of the site.	6	Omidvar, Liang, Cheng, Jianyu(2011), Jianyu(2012), Qiang
Size and Location	Accessibility	Refers to the easiness for getting to the shelter from the affected area.	7	Kilci, Omidvar, Liang, Cheng, Jianyu(2011), Jianyu(2012), Qiang
Size and Location	Proximity to homes of affected people	Shelter should be evenly distributed so that citizens can arrive there quickly before-in-after disaster.	7	Kilci, Omidvar, Liang, Cheng, Jianyu(2011), Jianyu(2012), Qiang
Size and Location	Infra structure conditions	Shelter areas should have electrical infrastructure, water supply, evacuation roads and sewage discharge.	3	Kilci, Qiang, Jianyu(2011)
Size and Location	Land drainage	Drainage of surface water and sewage is a key criterion especially where water is readily available.	3	Kilci, Omidvar, Jianyu(2012)
Size and Location	Soil permeability	Swift absorption of surface water by the soil is an important factor in site selection. It also influences the effectiveness of pit latrinnes.	3	Kilci, Omidvar, Liang
Size and Location	Physical layout and periphery configuration	For properly management of people entrance into the shelter.	2	Kilvi, Jianyu(2011)
Disaster risk reduction	Suitable distance from hazardous areas	Shelters should be far away from something dangerous such as huge buildings, flammable and explosive substances, hazardous chemicals, radioactive substances, high voltage transmission lines and secondary hazards.	6	Kilci, Omidvar, Liang, Qiang, Jianyu(2011), Jianyu(2012)
Disaster risk reduction	Geological hazards	Shelters should be keep away from seismic active fault,earthquake, landslide, collapse, debris flow, soil liquefaction and ground depression, etc.	5	Omidvar, Liang, Qiang, Jianyu(2011), Jianyu(2012)
Disaster risk reduction	Land slope	Land slopes steeper than 25 are considered to have a high risk of geo hazards, whereas those that dip at less than 5 are regarded as stable and secure.	5	Kilci, Omidvar, Liang, Qiang, Jianyu(2012)
Disaster risk reduction	Elevation	In subject of heavy rains, floods, and mudflow hazards, safe sites must be located at least 100m from river banks and terraces.	2	Qiang, Jianyu(2012)
Disaster risk reduction	Building protection standards	For available building which could be used as emergency shelters.	1	Qiang
Disaster risk reduction	Early warning availability	Having suitable early warning system for secondary disasters.	1	Qiang
Relief and rescue facilities	Water supply	Shelter should have water facilities which could meet drinking water, domestic water and fire water.	5	Kilci, Omidvar, Cheng, Qiang, Jianyu(2012)
Relief and rescue facilities	Suitable distance from medical centers	Shelters should be able to provide medical services. Therefore, such a site should be located as near as possible to medical centers.	5	Kilci, Omidvar, Cheng, Jianyu(2011), Jianyu(2012)
Relief and rescue facilities	Proximity to relief services	Shelter should be evently distributed so that citizens can receive relief items and services such as foods, tents, blankets, water and coverage of fire station.	5	Kilci, Omidvar, Liang, Cheng, Jianyu(2012)
Relief and rescue facilities	Communication service	Development of guiding signs and communication facilities such as telephones and radios etc.	2	Cheng, Qiang
Feasibility	Security and protection	It is recommended that affected people be settled at a reasonable distance from potentially sensitive areas, such as military installations, to ensure their protection and security.	5	Kilci, Omidvar, Liang, Qiang, Jianyt(2012)
Feasibility	Economic consideration	The selected site generally must be economically justifiable for the cost of establishment and costs after establishment.	3	Omidvar, Liang, Qiang
Feasibility	Land ownership	Ownership and usage rights of each shelter area should be predetermined and any necessary permission should be obtained.	2	Kilci, Omidvar
Feasibility	Previous land use	Land use before earthquake.	1	Omidvar
Environmental aspects	Environmental consideration	This criterion denotes seasonal variations and any related environmental hazards and diseases.	5	Omidvar, Liang, Jianyu(2011), Jianyu(2012), Qiang
Environmental aspects	Ecological recovery	The site should not be located near areas that are ecologically or environmentally protected or fragile.	2	Omidvar, Qiang
Environmental aspects	Vegetation	The site should provide sufficient ground cover for vegetation. Bushes, grass and trees, for example, supply shade and reduce dust and erosion.	2	Kilci, Omidvar
Environmental aspects	Possibility of agriculture	Soil conditions suitable for agriculture.	1	Omidvar
Social aspects	Culture, tradition and composition of population groups	Respecting to traditional customs and needs of people who may not be the same in composition, help managers to prevent some related problems between them and ensure that the shelter is functional and sustainable.	3	Omidvar, Liang, Qiang
Social aspects	Public opinion	CConsulting with local people is an important way to avoid or limit conflict over the location of shelter sites.	2	Omidvar, Qiang

Literature review, field investigations and interviews with the experts were done by the authors to explore the criteria. The interviews were done in various disciplines including geologists, environmental engineers, urban planners, rescue experts, local government officials, local residents, contractors, and social workers.

Five studies proposed model to apply several criteria for site selection. Analytical Hierarchy Process (AHP) was used by those studies to weight selected criteria for models.

GIS was also introduced by two articles as a tool for site selection and helping the emergency managers in decision making.

We categorized proposed criteria to the following six main categories: size and location, disaster risk reduction, relief and rescue facilities, feasibility, environmental and social aspects (table 2).

## Discussion and conclusion

In earthquakes, deciding how and where to shelter homeless people is a priority for relief agencies^4^. Disaster managers need to have proper criteria to select suitable sites for shelters after earthquakes.

Some criteria for shelter site selection have been introduced such as those by the Sphere project^12^. We found few papers presenting the criteria for shelter site selection after an earthquake. They were done to propose models for proper and systematic site selection on the base of some applicable criteria. All of them were done in areas with present risk of earthquake that shows the priority for this subject in earthquake prone areas.

Despite the focus of these papers on the earthquake, it seems that used criteria were not specific for earthquake. Considering the season, weather conditions, number of affected people, shelter type and model, and expected time for response and recovery phases could be important criteria in this way.

Used terminologies to present the sheltering type, were emergency and temporary. Although emergency and temporary sheltering are two separate phases in the ideal model, reviewing selected papers showed that all of them mean the temporary phase of this strategy.

We also must remember that “emergency sheltering” is used to cover both stages in most communities^7^^-^ 9. So in comparing the models or applying them, it is important to define the long term strategy for settlement.

Decisions about sheltering after earthquake is usually made in a limited time after the event without considering essential standards and based on individual reactions and the experiences of authorities. These decisions were presented as the most important drawback in this field^1^.

Meanwhile, having several criteria and trying to apply all of them is not possible and may lead to confusion in decision making^19^. In this situation, it is better to apply proper decision-making support tools.

However, decision-making support in this field raises many questions, some of them are hard to solve because they fall on the domain of cultural awareness, other questions are the ones in the technological domain that can be solved more easily^20^.

To develop objective models it is recommended to choose criteria among many available candidates by considering the three following principles: First, the number of them should be as few as possible. Second, they should be independent from each other. Third, they should be quantifiable in estimation^16^.

As such methods may limit managers in applying some valuable criteria, to overcome these limitations there are some related tools.

AHP and similar process represent application tools to standardize the involved criteria, and finally help choosing the best alternative decision to guarantee better performance^19^.

This process could be completed by proposing a comprehensive model. GIS could help users to apply more criteria in models and visually understand the model outputs.

A major goal of GIS planning is to implement models in which outputs support the decision process, reliably^21^. So users have a variety of information to use in critical decision makings.

Shelter site selection requires co­­-operating managers, rescue experts, environmental scientists, geologists, engineers, social workers, construction contractors and local residents, governmental and nongovernmental agencies^1^^,^ 16 ^,^ 18.

We suggest designing some studies in these groups with qualitative approaches to define specific applicable criteria for sheltering site selection after earthquakes.

Such criteria could be used to propose relevant models and decision support systems which are valuable tools to help managers for better decision making.

Having such models or systems and applying them in preparedness phase can help community planners to manage normal and emergency situations.

## Limitation

The main limitation of this review was that only English language papers included in the study as a systematic review. Therefore, we lost some of the relevant studies which were not in English language.

## Corresponding author

Ali Ardalan MD, PhD. 78, Italy Ave, Department of Disaster and Emergency Health, National Institute of Health Research, Tehran University of Medical Sciences, Tehran, Iran. Email: aardalan@tums.ac.ir

## Appendix 1

### PRISMA Checklist

Download PDF
